# Defining cure in multiple myeloma: a comparative study of outcomes of young individuals with myeloma and curable hematologic malignancies

**DOI:** 10.1038/s41408-018-0065-8

**Published:** 2018-02-28

**Authors:** Praful Ravi, Shaji K. Kumar, James R. Cerhan, Matthew J. Maurer, David Dingli, Stephen M. Ansell, S. Vincent Rajkumar

**Affiliations:** 1 Department of Internal Medicine,, Mayo Clinic, Rochester, MN USA; 2 Division of Hematology,, Mayo Clinic, Rochester, MN USA; 3 Division of Epidemiology, Department of Health Sciences Research,, Mayo Clinic, Rochester, MN USA; 4 Division of Biostatistics,, Mayo Clinic, Rochester, MN USA

## Abstract

Advances in therapy in recent years have led investigators to challenge the dogma that multiple myeloma (MM) is incurable. We assessed overall (OS) and progression-free survival (PFS) of young patients ( ≤ 50 years) with MM and compared outcomes with follicular lymphoma (FL), diffuse large B-cell lymphoma (DLBCL), and Hodgkin lymphoma (HL). All patients ≤ 50 years with newly diagnosed MM (*n* = 212), FL (*n* = 168), DLBCL (*n* = 195), and HL (*n* = 233) between 1 January 2005 and 31 December 2015 were included. Observed vs. expected survival was summarized by standardized mortality ratios (SMR). Compared to the background US population, excess mortality risk was seen at diagnosis in all four cancers, SMR 19.5 (15.2–24.5) in MM, 4.2 (2.3–7.2) in FL, 13.0 (9.2–18.4) in DLBCL, and 5.2 (2.6–9.3) in HL. We reasoned that cure would most likely occur in the first 3 years after diagnosis and be reflected by an overall survival probability similar to the background population. From the 36-month landmark, excess mortality risk was seen in MM (SMR 20.7 [14.7–28.3]) and FL (SMR 3.8 [1.5–7.8]), but not with DLBCL (SMR 3.1 [0.8–8.0]) or HL (SMR 0.9 [0.0–5.1]). MM patients have 20-fold excess mortality risk compared to the background population at diagnosis and at 3 years after diagnosis, suggesting that MM remains an incurable cancer.

## Introduction

In 1963, Easson and Russel, writing in the *British Medical Journal*, provided the first definition of ‘‘cure’’ in cancer, noting that it should “connote that in time—probably a decade or two after treatment—there remains a group of disease-free survivors whose annual death rate from all causes is similar to that of a normal population group of the same sex and age distribution^1^.” This was further extended by Frei and Gehan in 1971 to include that cure should be “unassociated with continuing morbidity from the disease or its treatment”^2^. The time-dependent concept of cure was further refined by Frei, who argued that the particular time point—typically between 1–5 years in most curable cancers—at which the plateau in disease-free survival ought to occur (to signify cure) varies depending on the disease kinetics of a particular tumor^3^.

Despite the simplicity of this concept, the use of the word cure in oncology is heterogeneous, with two thirds of published manuscripts containing that word in 2012 not meeting the above standard^4^. A poster child of this heterogeneity is found in the case of multiple myeloma (MM), a cancer where there has historically been a clash of treatment philosophies between disease cure and control^5^. In the past decade, the introduction of several new therapies has been shown to improve survival outcomes^6–9^ and there has been increasing adoption of autologous stem cell transplantation in eligible patients, which have both produced higher rates of complete response (CR)^10^. This has led investigators to challenge the traditional dogma that MM is an incurable disease^11–13^, and the use of the term ‘‘functional’’ or ‘‘operational cure’’ has gained traction to denote patients remaining in CR for a prolonged period of time^14,15^. However, most MM patients are on continuous suppressive therapy and face a persistent risk of relapse with no clear plateau in progression-free or overall survival (OS) demonstrated on intent to treat analysis of large clinical trials. Furthermore, unlike young patients with diffuse large B-cell lymphoma [DLBCL] and Hodgkin lymphoma [HL], who have a well-established probability of cure with normal or near-normal life span after a short (usually < 6 months) course of therapy, similar newly diagnosed MM patients cannot be assured of cure or normal life span, despite continuous or near-continuous therapy.

Based on these considerations, we sought to define survival outcomes and test the definition of cure in young ( ≤ 50 years) MM patients treated in the contemporary era, and compare outcomes with similarly aged patients with three other hematologic malignancies (follicular lymphoma [FL], DLBCL, and Hodgkin lymphoma [HL]), the latter two of which are widely considered to be curable cancers and meet accepted definitions of cure^2,16^. We specifically chose a cohort ≤ 50 years of age since this age group is likely to have few comorbidities, have access to and be able to tolerate the best possible treatments concurrently or sequentially, and thereby have the best opportunity to achieve ‘‘cure’’. Moreover, if the disease is curable, subsequent mortality in younger persons should be close to the general population. This eliminates the problem encountered in a disease affecting predominantly older adults, like MM, where curability is difficult to test due to deaths from competing causes.

## Methods

### Study cohort

The MM cohort comprised all patients under the age of 50 years who were diagnosed with MM between January 1, 2005 and December 31, 2015. Only those who were seen at our institution within 6 months of diagnosis were included. Patients in whom there was no information on follow-up (i.e., only attended for one clinic visit) were excluded, as well as those who had previously received anti-myeloma therapy for smoldering MM.

The lymphoma cohort consisted of patients under the age of 50 years with FL, DLBCL, and HL who enrolled in the SPORE Molecular Epidemiology Resource (MER) at our institution between January 1, 2005 and June 30, 2015. Descriptions of the MER cohort have been previously reported^17–20^. Briefly, all patients in the MER were prospectively enrolled within 9 months of diagnosis and then contacted systematically every 6 months for the first 3 years and then annually. Baseline clinical, laboratory, and treatment data were abstracted from medical records by using a standard protocol, and lymphoma diagnosis was reviewed by an expert hematopathologist. All progression, re-treatment, and deaths were verified through review of pathology and medical records.

### Outcomes

The primary outcomes of interest were OS and progression-free survival (PFS). OS was defined as the duration between date of diagnosis and death, or censored at the date of last follow-up. PFS in MM was defined as the duration between initiation of systemic treatment and disease progression, according to established criteria^21^. In the MER, an event-free survival definition (EFS) is utilized for PFS-style outcomes, due to the observational nature of the cohort. EFS in the MER is calculated as the time from diagnosis to progression/relapse, initiation of new therapy for disease progression or lack of efficacy, and death due to any cause. For purposes of nomenclature in this study, we will use PFS to describe the PFS (MM) and EFS (MER) results going forward. Patients without PFS events were censored at the date of last known disease status.

### Statistical analysis

Survival curves were generated using the Kaplan–Meier method and the log-rank test used to make comparisons between groups. Expected survival accounting for age and sex was generated in R by using the general United States (survexp.us) populations as the reference group^22^. Observed vs. expected OS was plotted by using a conditional approach and summarized by using standardized mortality ratios (SMRs) of observed to expected deaths^23^. Median follow-up was calculated using reverse Kaplan–Meier method. All tests were two sided with a *p*-value of < 0.05 considered to be significant. Statistical analysis was performed using SPSS v.22 (IBM Corp., Armonk, NY, USA) and Rv3.3.1.

## Results

The baseline demographics of all patients with MM (*n* = 212), FL (*n* = 168), DLBCL (*n* = 195), and HL (*n* = 233) included in this study are shown in Table 1. The median ages at diagnosis were 45, 42, 41, and 32 years, respectively. Patients received contemporary treatment regimes, with 92% of MM patients receiving a novel agent as part of induction chemotherapy and 52% having a hematopoietic stem cell transplant during their disease course. Median follow-up was 5.8 years (95% confidence interval [CI] 5.0–6.7) in patients with MM, 5.0 years (4.3–5.8) in FL, 4.9 years (4.4–5.4) in DLBCL, and 4.1 years (3.6–4.7) in HL patients.

**Table 1:** Baseline demographics of patients with MM, FL, DLBCL, and HL

	MM (*n* = 212)	FL (*n* = 168)	DLBCL (*n* = 195)	HL (*n* = 233)
Sex (%)				
Male	129 (61)	94 (56)	107 (55)	124 (53)
Female	83 (39)	74 (44)	88 (45)	109 (47)
Age, median (range)	45 (22–49)	42 (19–50)	41 (18–50)	32 (18–50)
Year of diagnosis (%)				
2005–2010	121 (57)	101 (60)	110 (56)	131 (56)
2011–2015	91 (43)	67 (40)	85 (44)	102 (44)
Disease stage (%)	ISS-1: 74 (35)	AA I–II: 35 (21)	AA I–II: 94 (48)	AA I–II: 133 (57)
	ISS-2: 46 (22)	AA III–IV: 133 (79)	AA III–IV: 101 (52)	AA III–IV: 94 (40)
	ISS-3: 48 (23)			N/A: 6 (3)
	N/A: 44 (21)			
Initial treatment (%)	PI-based: 96 (45)	Observation: 51 (30)	CHOP-based: 171 (88)	ABVD: 206 (88)
	IMID-based: 67 (32)	CHOP-based: 39 (23)	Other: 15 (8)	Other: 17 (7)
	IMID + PI: 31 (15)	Rituximab: 26 (15)	N/A: 9 (5)	N/A: 10 (4)
	Other: 12 (6)	R-CVP: 24 (14)		
	N/A: 6 (3)	Radiotherapy: 8 (5)		
		Other: 20 (12)		

*MM* multiple myeloma, *FL* follicular lymphoma, *DLBCL* diffuse large B-cell lymphoma, *HL* Hodgkin lymphoma, *ISS* International Staging System, *AA* Ann Arbor stage, *N/A* not available, *PI* proteasome inhibitor, *IMID* immunomodulatory agent, *CHOP* cyclophosphamide, doxorubicin, vincristine, prednisone, *R-CVP* rituximab, cyclophosphamide, vincristine, prednisone, *ABVD* doxorubicin, bleomycin, vinblastine, dacarbazine

### Survival outcomes and comparisons with the background population

**Fig. 1:**
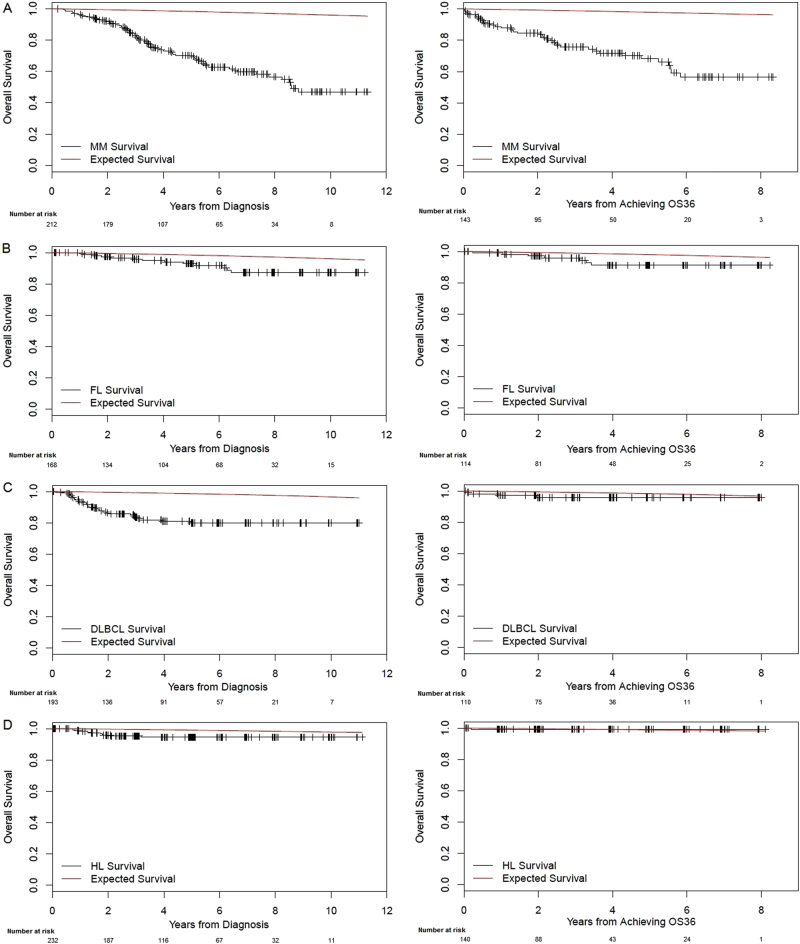
**Overall survival of patients with MM (1A), FL (1B), DLBCL (1C), and HL (1D) from diagnosis (left panel) and in those alive 3 years after diagnosis (right panel), compared with expected survival of the background population**

**Fig. 2:**
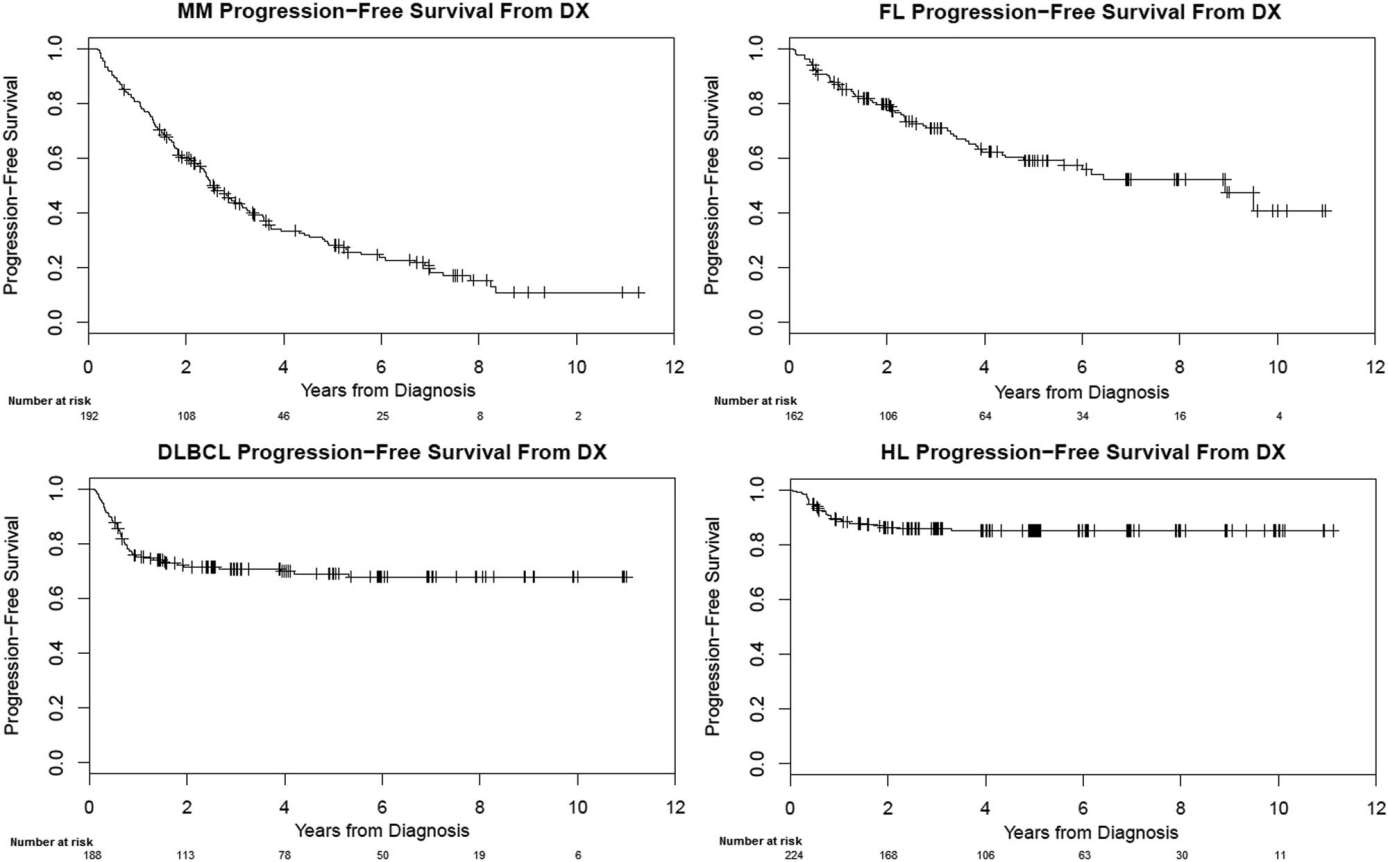
**Progression-free survival of patients with MM, FL, DLBCL, and HL**

Figure 1a–d (left panel) shows OS of patients from the time of diagnosis and expected survival of the background population. The 5-year OS was 70.2% (63.7–77.4) in patients with MM, 93.1% (88.7–87.6) in FL, 80.0% (73.9–86.6) in DLBCL, and 94.5% (91.4–97.8) in HL. PFS curves are shown in Fig. 2, with corresponding 5-year PFS rates of 28.3% (22.0–36.3), 59.1% (51.0–68.5), 69.0% (62.4–76.3), and 85.0% (80.3–90.0) in patients with MM, FL, DLBCL, and HL, respectively. OS and PFS curves for MM patients stratified by ISS are shown in Supplementary Fig. 1; there were no differences in either median OS (ISS-1, not reached; ISS-2, 7.8 years; ISS-3, 8.6 years; *p* = 0.461) or median PFS (ISS-1, 2.9 years; ISS-2, 2.6 years; ISS-3, 2.3 years; *p* = 0.586) between ISS sub-groups.

SMRs for patients at diagnosis are shown in Table 2. When comparing survival to the age- and sex-matched background population, a significant excess mortality risk was seen at the time of diagnosis in all four diseases, SMR 19.5 (15.2–24.5) in MM, 4.2 (2.3–7.2) in FL, 13.0 (9.2–18.4) in DLBCL, and 5.2 (2.6–9.3) in HL.

**Table 2:** Standardized mortality ratios (SMR) of patients with MM, HL, DLBCL, and HL at diagnosis, and at 36-month landmark (SMR-36)

	Diagnosis		36-month landmark	
	*N*	SMR (95% CI)	*N*	SMR-36 (95% CI)
MM	212	19.5 (15.2–24.5)	143	20.7 (14.7–28.3)
FL	168	4.2 (2.3–7.2)	114	3.8 (1.5–7.8)
DLBCL	193	13.0 (9.2–18.4)	110	3.1 (0.8–8.0)
HL	232	5.2 (2.6–9.3)	140	0.9 (0.0–5.1)

*MM* multiple myeloma, *FL* follicular lymphoma, *DLBCL* diffuse large B-cell lymphoma, *HL* Hodgkin lymphoma

### Thirty-six-month landmark analysis

We reasoned that patients would most likely be cured in the first 3 years after diagnosis and this would be reflected by an OS probability similar to the matched background population. Therefore, we performed a landmark analysis in patients alive 36 months after diagnosis (MM, *n* = 143; FL, *n *= 114; DLBCL, *n* = 110; HL, *n* = 140), with OS curves shown in Fig. 1a–d, right panel. The 3-year OS rates (from the 36-month landmark) were 75.6% (68.1–83.9) in patients with MM, 95.8% (91.9–100.0) in FL, 95.8% (91.9–100.0) in DLBCL, and 99.2% (97.8–100.0) in HL.

From the 36-month landmark time point, a significant excess mortality risk compared to the matched background population was observed in MM (SMR-36: 20.7 [14.7–28.3]) and FL (SMR-36: 3.8 [1.5–7.8]), but not in DLBCL (SMR-36: 3.1 [0.8–8.0]) or HL (SMR-36: 0.9 [0.0–5.1], Table 2).

## Discussion

Put simply, cure should be viewed as successful delivery of treatment(s) for a defined period of time with subsequent complete resolution of the disease. The patient should then expect to be able to enjoy a quantity and quality of life comparable with healthy counterparts once treatment has been completed. Although occasional patients with almost any disease may meet the definition of having achieved a cure, the point at which a disease as a whole can be considered curable requires that a predictable percentage of patients must achieve this state. This is best illustrated by the achievement of a plateau in OS (or disease-free survival) curves that oncologists appreciate in clearly curable cancers such as pediatric acute lymphoblastic leukemia or testicular cancer. An additional requirement for true cure is that the plateau persists despite therapy having been stopped. For example, are hypertension, diabetes, or human immunodeficiency virus (HIV) infection curable? Does ongoing molecular and morphologic remission of chronic myeloid leukemia with continuous imatinib therapy constitute a cure? If indefinite or lifelong administration of suppressive medication is needed, are we curing or controlling the disease?

MM is a disease that encapsulates the cure controversy. It has long been heralded as an incurable cancer. However, there have been advances in therapy, notably with autologous^24^ and allogeneic^25^ stem cell transplantation in the 1990s, and with newer immunomodulatory^6,7^ and targeted therapy^8,9^. Many patients are surviving beyond the 5-year mark that has traditionally been associated with ‘‘cure’’ in several malignancies. These have led some physicians to propose that MM has become a curable cancer^11^. Yet, there are two problems that make it difficult to determine the curability of diseases like myeloma. First, the disease affects older people in whom the disease can be controlled for many years, during which time they can die of other unrelated causes (competing risks). Thus it is hard to determine, even among a subset of these patients who are in CR, whether the disease was truly cured, or if a relapse would have occurred with time. Second, cure requires that therapy is given for a finite period of time, and demonstration that a proportion of patients remain free of relapse for a prolonged period of time. In diseases like MM where continuous therapy is administered, it is, therefore, difficult to ascertain cure.

In this paper, we studied young patients ( ≤ 50 years of age) with MM treated in the last 10 years, and compared their overall and expected survival to patients with FL, DLBCL, and HL. We show that almost all patients with HL and DLBCL alive 3 years after diagnosis are essentially cured—their subsequent survival is similar to the matched background population and there is an unequivocal plateau in the OS and PFS curves. In contrast, in MM, the 3-year landmark OS is markedly worse compared to the general population (Fig. 1), and even among patients who live many years, there is a relentless risk of relapse (Fig. 2). Hence, although young patients with MM have a 10-year OS of nearly 50%, they are typically not being cured. While there may be an apparent “plateau” in the OS and PFS curves, the numbers of patients at risk at these time points are small, and thus we have limited confidence that this signifies a true plateau.

In 2014, an update of the ‘‘total therapy’’ (TT) trials conducted at the University of Arkansas reported a ‘‘cure fraction’’ of almost 50% among all patients treated under the TT3a protocol^11^. This number, derived from a mathematical model, led them to conclude that MM had joined the “club of curable malignancies”. Although one cannot fail to be impressed by the Arkansas outcomes, two points must be appreciated. Firstly, comparison of the OS and PFS curves for MM with those for HL and DLBCL highlight that the level of ‘‘cure’’ achieved with MM is not that which is seen with the latter two cancers. This is supported by multi-institutional data showing that MM patients under the age of 50 lose an average of more than 25 years of life compared to healthy population controls^26^. Second, the treatment of MM (in our patients and with TT) incorporates a prolonged period of maintenance chemotherapy in contrast to HL or DLBCL, where therapy is limited to a defined number of cycles, with no evidence for a survival benefit arising from adjuvant or maintenance therapy^27^. Sustained CR in a subset of patients with MM enrolled in a very cumbersome clinical trial receiving years of continuous ongoing therapy is, in our opinion, not the same as showing a clear plateau in OS and PFS in patients treated with an intent to cure.

It is arguably true that there are some cancers that are inherently curable, whereas others are not, at least at the current time. Typically with the former, there are discoveries or advancements in therapy that produce paradigm shifts and blaze a trail towards cure. For example, testicular cancer was generally incurable until the discovery of cisplatin-based chemotherapy^28^. Similarly, the advent of MOPP therapy for HL led to a path towards cure for this and other lymphomas^29^. The use of cladribine has led to an effective therapy for hairy-cell leukemia^30^, while progressive advancements in chemotherapy and supportive care have produced a cure for childhood leukemia^31^. In the case of MM, there have been several impressive breakthroughs in treatment, including development of autologous and allogeneic hematopoietic cell transplantation, novel immunomodulatory agents and proteasome inhibitors, which have led to significant improvement in outcomes over the past two decades^32^. Further studies pursuing potentially curative strategies for early stage or smoldering disease are currently under way, and results are eagerly awaited^12^. However, our results suggest that talk about the disease being curable (at least in the majority of patients) is premature.

A major strength of our analysis is that we studied large cohorts of young patients treated at a major medical center with access to best available therapy (our 5-year OS rate of 70% is very similar to the 5-year rate of 74% seen with TT3a). Other strengths include the contemporary era of the study, and the sufficient follow-up (up to 10 years) that gave us a reasonable chance at assessing curability. The comparison of MM with three types of lymphoma, two of which are well-established as curable entities enables a more informed assessment of long-term survival and curability compared to viewing the disease in isolation. Finally, the purposeful inclusion of only young patients ( ≤ 50 years) enabled us to test curability in a cohort that, a priori, would be expected to have the greatest chance of being cured and in whom the likelihood of dying from other causes would be low.

Nevertheless, there are certain limitations that need to be acknowledged. First, this was a single-center retrospective study from a tertiary referral center, which may limit the generalizability of the results. Secondly, although our patients had access to and received the best treatments available in the United States, the regimens used were heterogeneous. We also did not assess particular subsets of MM patients (such as those achieving MRD negativity) in whom the possibility of a cure may exist; however, we did stratify MM patients by ISS and did not find that patients with lower stage disease showed a greater propensity to be cured (Supplementary Fig. 1). Finally, we were unable to capture downstream effects from treatment (such as secondary malignancies), which is an important component of the original cure definition^2^, or outcomes beyond 10 years.

In conclusion, we found that survival outcomes for young patients with MM are strikingly different from those seen with similarly aged patients with HL and DLBCL. Young patients who survive 3 years with DLBCL and HL achieve true cure with an OS similar to the background population. In contrast, patients with MM and FL continue to relapse over time, with a significantly inferior OS to the background population. As the old adage goes, a picture is worth a thousand words: Figs. 1 and 2 illustrate how OS and PFS curves of curable diseases (DLBCL and HL) look like in a population that has the best chance of being cured and with a low probability of dying from other causes. Readers and investigators can determine for themselves if MM (or FL) appear to be curable diseases. We are confident that when we see cure, we will know it.

## Electronic supplementary material

Supplementary Figure 1
