# Supplementary material for: Defining cure in multiple myeloma: a comparative study of outcomes of young individuals with myeloma and curable hematologic malignancies
Source: Blood Cancer J. 2018 Feb 28;8(3):26. doi: 10.1038/s41408-018-0065-8 (PMC5849889; doi:10.1038/s41408-018-0065-8)
Supplement: Supplementary file 1 — Supplementary Figure 1 [file 41408_2018_65_MOESM1_ESM.docx]

**Supplementary Figure 1: OS (1A) and PFS (1B) of MM patients, stratified by International Staging System (ISS)**

**Figure 1A:**


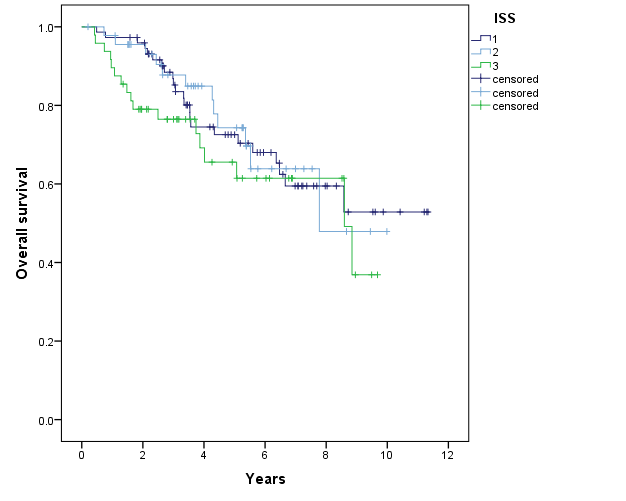


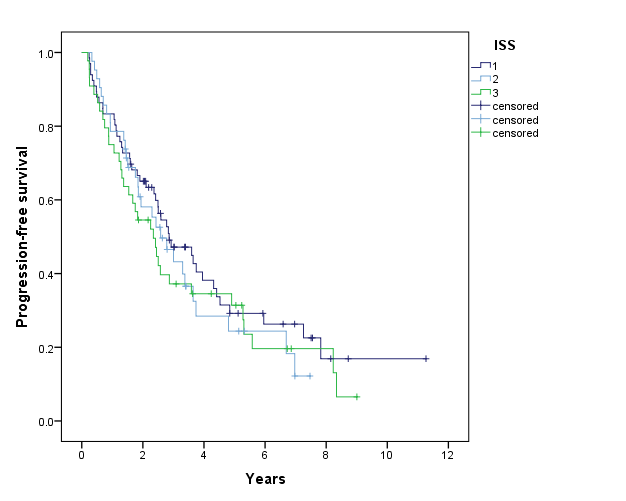
**Figure 1B:**
